# Current Challenges in Deciphering Sutton Nevi—Literature Review and Personal Experience

**DOI:** 10.3390/jpm11090904

**Published:** 2021-09-09

**Authors:** Roxana Nedelcu, Alexandra Dobre, Alice Brinzea, Ionela Hulea, Razvan Andrei, Sabina Zurac, Mihaela Balaban, Mihaela Antohe, Lorena Manea, Andreea Calinescu, Anastasia Coman, Florentina Pantelimon, Adina Dobritoiu, Catalin Popescu, Raluca Popescu, Elena Balasescu, Daniela Ion, Gabriela Turcu

**Affiliations:** 1 General Medicine, Carol Davila University of Medicine and Pharmacy, 050474 Bucharest, Romania; roxanaioana.nedelcu@yahoo.com (R.N.); brinzeaalice@gmail.com (A.B.); dr.ionelahulea@yahoo.com (I.H.); sabina_zurac@yahoo.com (S.Z.); dr.antohemihaela@gmail.com (M.A.); lor.manea@gmail.com (L.M.); andreea.calinescu0202@yahoo.com (A.C.); anastasia.hodorogea@gmail.com (A.C.); catalin.m.popescu@gmail.com (C.P.); rlc.popescu@gmail.com (R.P.); elena_balasescu@yahoo.com (E.B.); danielaion7@ymail.com (D.I.); dr.gabriela.turcu@gmail.com (G.T.); 2 Derma 360 Clinic, 11273 Bucharest, Romania; mihaela.cernat@gmail.com (M.B.); florentina.pantelimon@gmail.com (F.P.); adina_dobritoiu@yahoo.com (A.D.); 3 National Institute for Infectious Diseases, Outpatient Clinic, 021105 Bucharest, Romania; 4 Department of Pathology and Dermatovenerology, Colentina Clinical Hospital, 020125 Bucharest, Romania; razvan.andrei.13@gmail.com; 5 Synevo Medical Laboratory, 014192 Bucharest, Romania; 6 Department of Dermatovenerology, Centre Hospitalier Régional D’orléans, 45100 Orléans, France

**Keywords:** immune response, inflammation, Sutton nevi, halo nevi, skin tumor

## Abstract

Halo nevi, known as leukoderma acquisitum centrifugum, Sutton nevus, leukopigmentary nevus, perinevoid vitiligo, or perinevoid leukoderma, together with vitiligo and melanoma-associated hypopigmentation, belong to the group of dermatoses designated as immunological leukodermas. The etiology and pathogenesis of halo nevi has not been fully elucidated. There are several mechanisms through which a lymphocytic infiltrate can induce tumoral regression. In this review, we aimed to update the knowledge about Sutton nevi starting with the clinical appearance and dermoscopic features, continuing with information regarding conventional microscopy, immunohistochemistry, and the immunological mechanisms responsible for the occurrence of halo nevi. We also included in the article original unpublished results when discussing dermoscopic, pathologic and immunohistochemical results in halo nevi. Sutton nevi are valuable models for studying antitumor reactions that the human body can generate. The slow and effective mechanism against a melanocytic skin tumor can teach us important lessons about both autoimmune diseases and anticancer defenses.

## 1. Introduction

*Leukoderma acquisitum centrifugum*, later known as *Sutton nevus* or *halo nevus*, appeared in 1916 in the writings of Richard Lightburn Sutton [1,2]. The recognition of Dr. Richard Lightburn Sutton’s work from whom the clinical entity takes its name has its root in describing and observing for the first time the disease in two of his patients [1,2]. Sutton described two cases of peculiar brown maculo-papules, but he considered the lesions to be clinical variants of vitiligo. He introduced his cases of abnormal pigmentation with a review of the cellular origins of pigmentation and vitiligo. It was only later that John H. Stokes made the correlation of *leukoderma acquisitum centrifugum* with melanocytic nevi [3].

With no gender or racial predilection, Sutton nevi are found in approximately 1% of young adults. The mean age at onset is thought to be 15 years. It is unusual and a sign of suspicion if a depigmentation around a nevus is found in an older patient [4,5].

The most common sites for Sutton nevi are the back, followed by head and neck [6,7]. The halo phenomenon can develop around preexisting junctional or compound nevi. [8].

Attempts have been made to better understand the natural evolution of halo nevi, by describing and didactically dividing the disease into four stages [8,9]: Stage I corresponds to the presence of the characteristic depigmented rim around the nevus; Stage II, with loss of pigment within the central nevus surrounded by a depigmented rim; Stage III is described by the presence of a circular area of depigmentation with the total disappearance of the nevus; Stage IV corresponds to the repigmentation phase, with normal appearing skin. The changes can develop for over 10 years.

## 2. Why Measure the Diameter?—The Average Size of Halo Nevi, the Relationship between the Halo and the Nevus

The stages mentioned above are variable with every patient and natural evolution of Sutton nevi has not yet been fully deciphered. The presence of halo depigmentation correlates with the onset of nevus regression, evolving towards total resorption of the melanocytic lesion with the possibility of repigmentation after many years. Complete regression of the central nevus has been reported in over 50% of cases [10]. Even if it is known that the halo phenomenon has four clinical stages of evolution, an unusual clinical evolution with repigmentation of the nevus component was also reported [11,12].

The size of the hypomelanotic halo varies and the most frequently reported values in the literature were between 0.5 and 5 cm [13]. At present, there are few data in the literature related to the factors that influence the diameter of the halo, the significance of this diameter and the possible correlations between the diameter of the nevus and the diameter of the halo. Rongioletti et al. were among the few authors who studied these possible correlations. They calculated a correlation coefficient and showed the existence of a linear and highly significant direct link between these two parameters. Thus, the larger the nevus, the larger its halo. They were also the ones who described the “melanocyte antigenic unit” (melanocyte antigenic unit—MAU). It consists of melanocytes with radial disposition from the central melanocytic lesion, having the same phenotype and being functionally dependent on the central nevus. This concept provides a valid explanation for the etiopathogenesis of the recurrent melanocytic nevus and also seems to provide useful information in understanding the link between melanoma margins and tumor thickness [14]. Further studies are still needed to explore in more detail the connection between the halo and the thickness of the central nevus.

## 3. Dermoscopic Pattern—Same Look as in Common Nevi?

For the last few years, dermoscopy has been shown to be one of the most helpful tools used to diagnose and monitor skin tumors. Despite the fact that the Sutton nevus is a benign lesion, the diagnostic process is often challenging, because it may present clinical, dermoscopical and histopathological structures that resemble melanoma. Furthermore, dermoscopic features that characterize this type of nevi have not yet been described in detail, so we will present you hereunder a comparison between the actual data from the literature and our own personal findings.

We retrospectively analyzed 32 digital dermoscopic images of Sutton nevi from 17 patients. The diagnosis was made by experienced dermatologists and was mostly based on clinical and dermoscopic examination. All nevi were checked with a hand-held DermLite DL4W dermoscope and stored afterwards as digital images in a computer database. Of the 17 patients, 11 (64.7%) were women and six (35.3%) were men. The mean age at presentation was 26.05 years (median 29; range 9–50), somewhat higher than previous studies (18.2%; 22.4%) [15,16]. The most frequent Fitzpatrick skin phototype we found was type III (15/17; 88.22%), followed by type II (2/17; 11.8 %). Between 25% and 50% of people with Sutton nevi have been described with more than one nevus [8]. In our group, the ratio of solitary to multiple nevi was 11:6, even though most occurred as single lesions. Out of the 32 halo nevi, 20 (62.5%) were located on the chest, five (15.6%) were located in the lumbar region, three (9.4 %) were located on the abdomen, two (6.62%) in the head region and two (6.62%) on upper extremities. Indeed, a predilection for the trunk has been reported in the literature [13].

The appearance of the nevi varied between flat (*n* = 25, 72.1%), elevated (*n* = 4, 12.5%) and central elevated (*n* = 3, 9.4 %). The first dermoscopic aspects that we analyzed were size and color. Similarly to previously published studies, the mean diameter of the central nevus was 5.22 mm (range 3–10) [8]. Most were light brown (*n* = 11), but we also observed brown (*n* = 6), pink (*n* = 2), or multicolored (*n* = 3) nevi. Additionally, nine were fully regressed with a residual white halo and one was fully regressed with total repigmentation of the skin.

**Figure 1:**
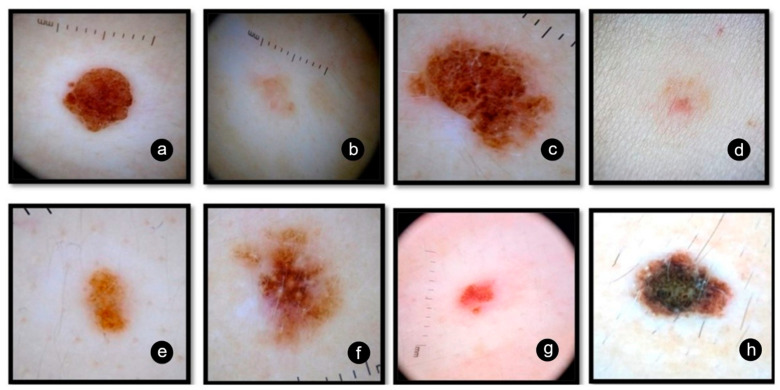
(**a**) Sutton nevus with symmetrical white rim; (**b**) Sutton nevus with asymmetrical white rim; (**c**) Sutton nevus with regional white patch; (**d**) Sutton nevus with no depigmentation area; (**e**) Complex pattern: reticulo-globular; (**f**) Complex pattern: globular-homogeneous with blue white veil and peppering; (**g**) Homogeneous pattern with coma-like vessels; (**h**) Multicomponent pattern: inverse network with peripheral globular-homogeneous.

Next, we analyzed the halo of the nevi and we observed a particularity. According to Sutton and the existing literature, the surroundings of the central nevus should be a completely well-circumscribed depigmentation area [13]. In our case, this classical uniformly annular halo was not seen in all nevi. For 21 (65.6%) of them, we observed a symmetrical white rim (Figure 1a); for seven (21.9 %) an asymmetrical white rim (Figure 1b); whereas two (6.25%) had just a local white patch (Figure 1c) and two (6.25%) nevi lacked any halo or depigmented area (Figure 1d). A recent study described two other particular types of halo: one with visible telangiectasis and a pinkish surrounding halo [15]. The mean diameter of Sutton nevi for our group was 12.06 mm (range 5–19 mm).

In terms of global structure, out of the 32 nevi, 16 (50%) were symmetrical, six (18.75%) were asymmetrical and 10 (31.25%) were fully regressed. Of the regressed nevi, nine had a residual white halo and one was completely repigmented. We also found out that 14/32 (43.75%) nevi had developed poliosis. Generally, the diagnosis of these cases is clinical, but given the several worrisome dermoscopic findings, we had to excise three nevi for histopathological diagnosis (Figure 1d,f).

In Table 1, we present a synopsis of the dermoscopic pattern we found in our group of patients. Half of the nevi had a uniform distribution of pigmentation, 31.25% had no pigmentation, 9.4% were multifocal hyper/hypopigmented and fewer were the central hyperpigmented annular type (6.25%) and eccentric hyperpigmented type (3.1%). Homogeneous (62.5%) and globular (37.5%) patterns were most frequently encountered; findings that support the established literature [17]. Reticular pattern was seen in three nevi (9.4 %), one of which had diffuse typical network, another had inverse network and the last one had patchy peripheral typical network. In regard to structureless pattern, 18 nevi had just one homogeneous color and two had two homogeneous colors. Of 12 nevi with globular pattern, seven had regular globules, four had irregular globules and one had cobblestone pattern.

We could only establish the global pattern for 22 nevi, since 10 were fully regressed. Complex patterns were most common (11/32); two nevi were reticulo-globular and nine were globular-homogeneous (one of them had also two local regression structures; blue white veil and blue pepper-like granules). Of the 32 nevi, 10 were homogeneous (31.25%), five of which also presented local features (four with coma-like vessels and one with blue pepper-like granules). The least frequent pattern was the multicomponent one (1/32), which presents a combination of globular, reticular and homogeneous features.

**Figure 2:**
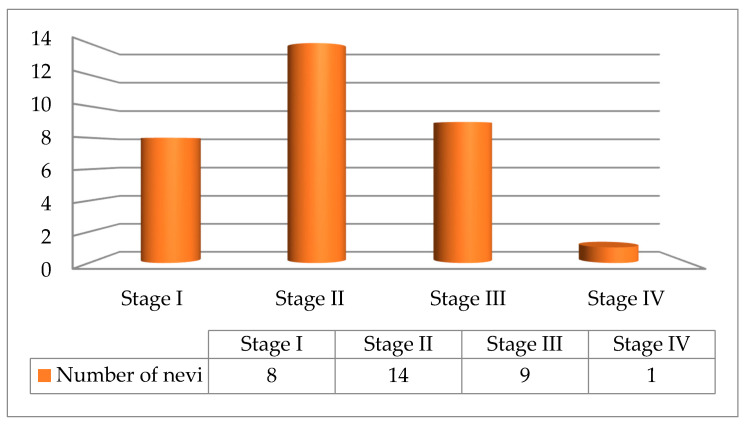
Stages proportion in our investigated group with Sutton nevi.

After analyzing the dermoscopic pattern of all nevi, we were able to categorize them into four stages. The proportion of each nevus stage was represented in Figure 2.

**Figure 3:**
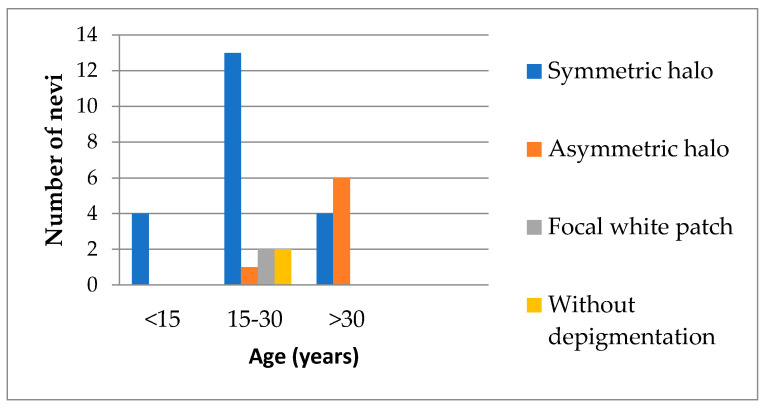
Types of depigmented rim depending on age.

Finally, it must be underlined that depigmentation may occur around the lesion, within it as focal white patches, at distance from halo nevi [18], or it may not be visible at all. The different types of achromatic halo that depend on age are shown in Figure 3. It is worth noting that all patients below the age of 15 had nevi with a symmetrical halo (4/4), a proportion which decreased for patients aged between 15 and 30; they also presented nevi with focal white depigmentation (*n* = 2), with asymmetrical halo (*n* = 1) and without any depigmentation (*n* = 2). In the group of patients aged over 30 the ratio of symmetrical (4/10) and asymmetrical (6/20) halo was reversed.

**Figure 4:**
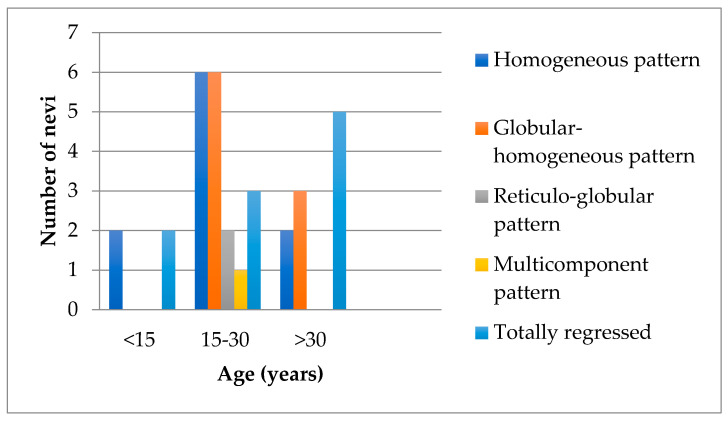
Dermoscopic patterns depending on age.

Moreover, Figure 4 shows the occurrence of dermoscopic patterns depending on age. We noticed that the number of regressed nevi increases with age. Nevertheless, atypical Sutton nevi were especially found in the group of patients aged 15 to 30 years. Two of them had a focal depigmentation area (Figure 1c,f) and one did not present with any patch of depigmentation (Figure 1d). Additionally, two nevi had local regression structures such as blue white veil and peppering (Figure 1d,f) and one had a multicomponent concentric pattern (Figure 1h). Even though dermoscopy allows a more accurate diagnosis of melanocytic skin lesions, when evaluation emphasizes atypia, a subsequent histopathologic diagnosis should be considered [19]. We want to highlight the fact that sometimes it is really hard to distinguish between halo nevus from an area of melanoma regression, and the patient age should not be the main criteria when deciding to perform a biopsy [16]. Our analysis is limited by the rarity of this dermatological entity and implicitly, the low number of patients included in this study.

## 4. The Microscopic Features—What Matters in Atypical Tumors?

From a clinical point of view, halo nevi develop in several stages, depending on which the dermoscopic appearance is different, as we detailed above. Histologically, four forms are described without always having a dermoscopic correspondence: (a) inflammatory, (b) noninflammatory, (c) halo nevus without halo, (d) halo dermatitis around a melanocytic nevus, and a classification focused on inflammatory infiltrate is presented below [20,21]. The clinical correlation is very important because some nevi may demonstrate marked inflammation, but, clinically, no halo is visible, and other, noninflammatory halo nevi, may demonstrate a halo, but histologically no inflammatory infiltrate.

The diagnosis of halo nevus is usually considered an easy one, based only on the clinical aspect. However, atypical presentations, at the beginning of the depigmentation or repigmentation can be challenging even on dermoscopy and reflectance confocal microscopy and the lesion needs to be excised to rule out melanoma [22]. Bruges et al. estimated that atypical halo nevi represent about 1.4% of all atypical benign nevi excised [22]. Regression structures identified on dermoscopy may also generate histopathological controversy [23].

Halo phenomenon refers to the presence of a dense lymphohistiocytic infiltrate that can be associated with congenital melanocytic nevi, Spitz nevi, congenital giant nevocellular nevi, balloon cell nevi, or atypical nevi, and it can be prominent even before the clinical evidence of the depigmentation [24,25]. The inflammatory infiltrate has a different density, composition and distribution in different stages of the regression. In 1994, Akasu et al. characterized four stages of the process and more the one can be present at a time in a single lesion: In stage I (pre-regression) intact nests can be found and no atypia, and the inflammatory infiltrate (CD3 positive more than 50%, CD4 positive approximate 25 %, few Langerhans cells) surrounds nevus nests. Stage II (early regression) is defined by the presence of nests with ragged edges mild nuclear atypia and the inflammatory infiltrate (similar lymphocytes distribution and increased number of S-100 protein-positive Langerhans cells) surrounds and invades nevus nests. Stage III (late regression) is characterized by single or ill-defined clusters, with nuclear atypia and a dense infiltrate within nevus clusters (numerous enlarged FXIII_a_—positive cells and numerous S-100 protein—positive epidermal Langerhans cells). In Stage IV (complete regression), there are no nevus cells, moderate dermal inflammatory infiltrate, along with flattening of the epidermis and neovascularization in the superficial dermis [26].

Studies have shown that halo nevi exhibited a broad spectrum of atypia (mild, moderate, severe), so halo nevi should not be regarded as a single clinicopathological entity [27].

The etiology and pathogenesis of halo nevi has not been fully elucidated. Two theories have been discussed as pathogenic mechanism: the antibody theory and the cytotoxic T cell response. The antibody theory has been downplayed, because it has been shown that activated and cell proliferating lymphocytes disappeared after excision of the halo nevus [28]. Mononuclear infiltrates in halo nevus consist of about 80% of T cell lymphocytes, mainly CD8 + T cell. Most T cells are positive for granzyme B, perforin, and Fas ligand, illustrating their cytotoxic activity [29].

Immunohistochemical studies suggest a stronger cytotoxic response (higher prevalence of granzyme) in halo nevi compared with melanoma, and a higher immune-regulatory mechanism (higher prevalence of PD1) in halo nevi [30]. Additionally, the number of CD8 + cells overcome the number of CD4 + T cells in halo nevi.

The most challenging differential diagnosis of halo nevi is malignant melanoma with regression. There are some architectural differences such as: the proliferating cell type, the distribution and type of the inflammatory infiltrate, and the effect of the tumor cells destruction. In halo nevi, the atypical cells are in the junctional nests and upper portion of the nevus with maturation toward the base of lesion. The inflammatory infiltrate in early regression stages of melanoma may look similar to the one in halo nevi. FOXP3 regulatory T cells are highly expressed in early stages of halo nevi [31], while in regression areas of melanoma, a small number of regulatory T cells was described [31,32]. The inflammatory cell infiltrate is distributed in a band-like pattern, symmetrically around the nevus, while in regressing primary melanoma it is asymmetric, surrounding only some segments of the tumor [33].

The autoimmune phenomena may induce reactive atypia and affect the architecture of the nevocytes, making the differential diagnosis even more challenging. Traditionally, regression in nevi is not accompanied by fibrosis, contrary to melanoma where there are thick collagen bundles. Some studies have demonstrated that in the late stages of regression, nevi can be thin and delicate collagen bundles suggesting a feedback mechanism or balance between apoptotic factors and tolerogenic factors [34]. Although this difference is not completely understood, antifibrotic cytokine tumor necrosis factor (TNF-α) was significantly highly expressed in halo nevus than in melanoma, while fibrogenic cytokines were more frequently expressed in melanoma [33].

HMB45 staining can be useful in distinguishing the two entities. Typically, in halo nevus, lesional melanocytes are HBM45-positive in the superficial dermis and HMB45-negative in the deep dermis. On the other hand, in melanoma, HMB45 staining can be either irregular or diffusely positive throughout the lesion or can be negative [25,35]. Atypical distribution of HMB45 positivity was described in halo nevi and more accurate tests are necessary to descriminate between halo nevus and melanoma [25].

In the recent years, complementary preferentially expressed antigen in melanoma (PRAME) was used to help differentiate benign from malignant melanocytes, as it is rarely expressed in nevi [36].

**Figure 5:**
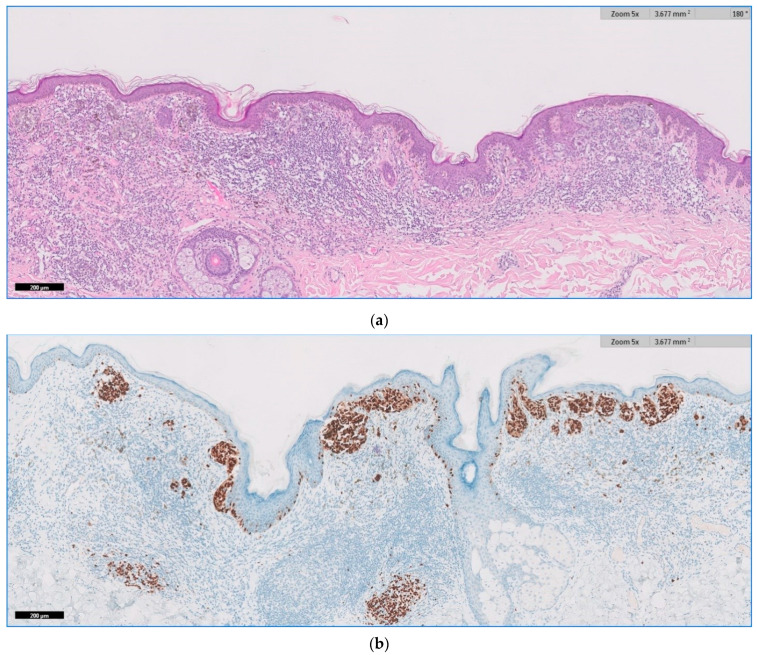
(**a**) HE and (**b**) SOX10. Melanocytic lesion with irregular distribution of nests at the dermal–epidermal junction and dense chronic inflammation (halo type) within the dermis, partially obscuring the dermal component.

**Figure 6:**
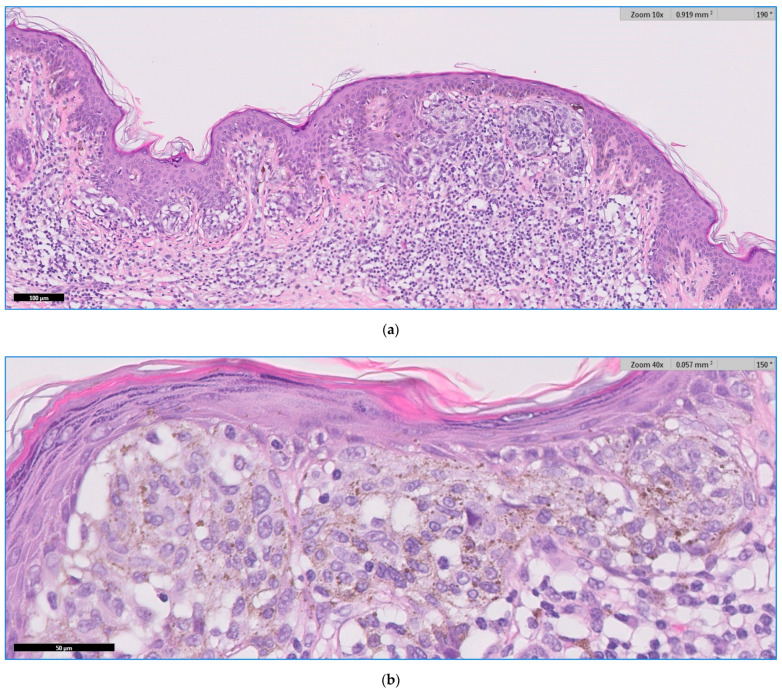
(**a**,**b**) HE. Fusion of junctional melanocytic nests with low-grade dysplasia.

**Figure 7:**
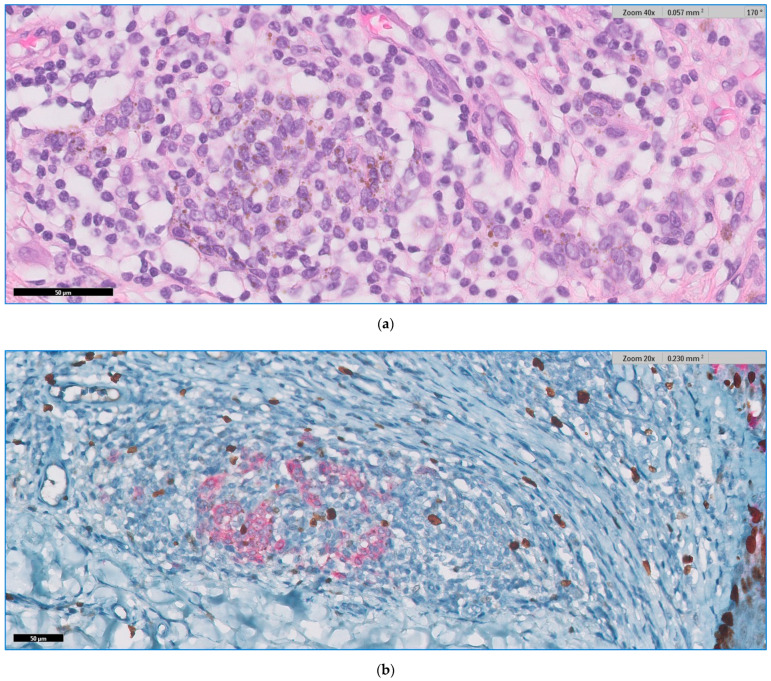
(**a**) HE and (**b**) Ki67-Mart1 dual stain. The halo phenomenon showing lymphocytic inflammation intermigled with benign nevus cells.

We present below the unpublished histopathological and immunohistochemical aspects (Figure 5, Figure 6 and Figure 7) from the case of a 29-year-old patient, with atypical features, clinically and dermoscopically (Figure 1f).

## 5. Association with Diseases—What to (Not) Investigate?

There are many diseases that have been described in individuals with Sutton nevi, such as vitiligo, thyroid diseases, and neoplasia [18,19,37,38]. Among them, vitiligo is the most frequently seen [19]. The association with malignant diseases needs further investigation.

Even though studies have focused on finding out whether patients with halo nevi are susceptible to developing vitiligo, a consensus is lacking. When associated with vitiligo, halo nevi tend to appear earlier in life [18,37]. In other words, the lower the age of the patient with halo nevi, the greater the risk of developing vitiligo. The idea that multiple halo nevi rather than a single lesion associate with vitiligo was suggested by many authors in studies involving children [39] and adults [37]. On the other hand, van Geel et al. considered that the risk of vitiligo decreased when the number of halo nevi was greater than three [18].

Zhou et al. demonstrated that patients with halo nevi and Koebner phenomenon had a greater risk of vitiligo [37]. Moreover, a patient with halo nevi has an increased risk of contracting vitiligo if a member of his family has vitiligo [37].

Great significance was given to studies that involve pediatric patients with vitiligo and halo nevi. A study showed that halo nevi were present in more than a quarter of children with vitiligo [40]. Many of them supported the idea that pediatric patients with vitiligo were more affected by halo nevi than adults with the same disease [39]. The scientific work conducted by Cohen et al. showed that male children with vitiligo were more affected by halo nevi [40]. Moreover, the pediatric patients that had only vitiligo were younger at the moment of the diagnosis than the patients who had both diseases at the same time [40]. A higher incidence of halo nevi was observed in children with generalized vitiligo than in those with more localized forms (segmental and focal) [40]. The idea that children with generalized vitiligo were more prone to develop halo nevi was highlighted in other articles [41].

Autoimmune diseases, especially Hashimoto’s thyroiditis, were more frequently seen in patients with both halo nevi and vitiligo when compared to halo nevi without vitiligo [37]. Considering the fact that vitiligo can be associated with a thyroid pathology, the authors suggested that blood tests should be recommended for patients with Sutton nevi thyroid, in order to estimate the risk of vitiligo [37].

Halo nevi can also appear as an adverse effect of a treatment. Immunotherapy-induced halo nevi was described in patients treated with atezolizumab [42] and ipilimumab [43].

As far as it is known in the literature, the presence of Sutton nevi does not carry an additional risk for the appearance of primary melanoma. However, the link between neoplasia and eruptive Sutton nevi was discussed by Lorentzen in a case series study, where 16 patients with eruptive halo nevi were followed for six years [38]. All the patients were adults and most of their nevi (>80%) had become Sutton nevi [38]. No immunotherapy was administered prior to the appearance of the lesions. During this period, eight patients developed a malignant disease. There was an increased incidence of melanoma (955 times higher than expected) and cancer overall (papillary thyroid cancer, neuroendocrine lung tumor, lung metastases from melanoma) [38].

## 6. Key Antimelanocyte Immune Reaction Lesson

The etiology and pathogenesis of halo nevi has not been fully elucidated. There are several mechanisms through which a lymphocytic infiltrate can induce tumoral regression. The marked inflammatory infiltrate seen in the histopathological examination suggests an immune-based mechanism. Two theories have been discussed as pathogenic mechanism: the antibody theory and the cytotoxic T cell response. The antibody theory has been downplayed, because it has been shown that activated and cell proliferating lymphocytes disappeared after excision of the halo nevus [28]. Mononuclear infiltrates in halo nevus consist of about 80% of T cell lymphocytes, mainly CD8 + T cell. Most of the T cells are positive for granzyme B, perforin, and Fas ligand, illustrating their cytotoxic activity [29].

In this review, we aimed to update the knowledge about Sutton nevi starting with the clinical appearance, the dermoscopic features, continuing with information regarding conventional microscopy, immunohistochemistry, and the immunological mechanisms responsible for the occurrence of halo nevi. We also included in the article original unpublished results when discussing dermoscopic, pathologic and immunohistochemical results in halo nevi.

Sutton nevi are valuable models for studying the antitumor reactions that the human body can generate. The slow and effective mechanism against a melanocytic skin tumor can teach us important lessons about both autoimmune diseases and anticancer defenses.

## Figures and Tables

**Table 1:** Dermoscopic features of our investigated patient group with Sutton nevi.

Global Structure	
Symmetric	16/32 (50%)
Asymmetric	6/32 (18.75%)
Regressed	10/32 (31.25%)
Size- mean, median, min, max (mm)	5,22, 5, 3, 10
**Distribution of Pigmentation**	
Uniform	16/32 (50%)
Centralhyperpigmentedannular type	2/32 (6.25%)
Eccentric hyperpigmented type	1/32 (3.1%)
Multifocalhyper/hypopigmentedtype	3/32 (9.4 %)
No pigmentation	10/32 (31.25%)
**Structural Patterns**	
**Globular**	**12/32** (**37.5%**)
Regular	7/32 (21.9 %)
Irregular	4/32 (12.5%)
Cobblestone	1/32 (3.1%)
**Network**	**3/32** (**9.4 %**)
Typical	1/32 (3.1%)
Inverse	1/32 (3.1%)
Patchy Peripheral	1/32 (3.1%)
**Homogeneous**	**20/32** (**62.5%**)
One Color	18/32 (56.25%)
Two Colors	2/32 (6.25%)
**Regression**	**25/32** (**59.4 %**)
Partial	15/32 (46.9 %)
Total	10/32 (31.25%)
**Local Features**	
Vascular Pattern—Coma-Like Vessels	5/32 (15.6%)
Poliosis	14/32 (43.75%)
**Regression Structures**	
Blue White Veil	1/32 (3.1%)
Peppering	2/32 (6.25%)
**Surrounding Halo**	
Symmetric	21/32 (65.6%)
Asymmetric	7/32 (21.9 %)
Regional	2/32 (6.25%)
Without	2/32 (6.25%)
Size- mean, median, min, max (mm)	12.06, 12, 5, 19
